# Prevention literacy: community-based advocacy for access and ownership of the HIV prevention toolkit

**DOI:** 10.7448/IAS.19.1.21092

**Published:** 2016-09-30

**Authors:** Richard G Parker, Amaya Perez-Brumer, Jonathan Garcia, Kelly Gavigan, Ana Ramirez, Jack Milnor, Veriano Terto

**Affiliations:** 1 Department of Sociomedical Sciences, Mailman School of Public Health, Columbia University, New York, NY, USA; 2 Associação Brasileira Interdisciplinar de AIDS (ABIA), Rio de Janeiro, Brazil; 3 College of Public Health and Human Sciences, Oregon State University, Corvallis, OR, USA; 4 Department of Population and Family Health, Mailman School of Public Health, Columbia University, New York, NY, USA

**Keywords:** prevention literacy, treatment literacy, health literacy, HIV/AIDS, HIV prevention, AIDS expertise

## Abstract

**Introduction:**

Critical technological advances have yielded a toolkit of HIV prevention strategies. This literature review sought to provide contextual and historical reflection needed to bridge the conceptual gap between clinical efficacy and community effectiveness (i.e. knowledge and usage) of existing HIV prevention options, especially in resource-poor settings.

**Methods:**

Between January 2015 and October 2015, we reviewed scholarly and grey literatures to define treatment literacy and health literacy and assess the current need for literacy related to HIV prevention. The review included searches in electronic databases including MEDLINE, PsycINFO, PubMed, and Google Scholar. Permutations of the following search terms were used: “treatment literacy,” “treatment education,” “health literacy,” and “prevention literacy.” Through an iterative process of analyses and searches, titles and/or abstracts and reference lists of retrieved articles were reviewed for additional articles, and historical content analyses of grey literature and websites were additionally conducted.

**Results and discussion:**

Treatment literacy was a well-established concept developed in the global South, which was later partially adopted by international agencies such as the World Health Organization. Treatment literacy emerged as more effective antiretroviral therapies became available. Developed from popular pedagogy and grassroots efforts during an intense struggle for treatment access, treatment literacy addressed the need to extend access to underserved communities and low-income settings that might otherwise be excluded from access. In contrast, prevention literacy is absent in the recent surge of new biomedical prevention strategies; prevention literacy was scarcely referenced and undertheorized in the available literature. Prevention efforts today include multimodal techniques, which jointly comprise a toolkit of biomedical, behavioural, and structural/environmental approaches. However, linkages to community advocacy and mobilization efforts are limited and unsustainable. Success of prevention efforts depends on equity of access, community-based ownership, and multilevel support structures to enable usage and sustainability.

**Conclusions:**

For existing HIV prevention efforts to be effective in “real-world” settings, with limited resources, reflection on historical lessons and contextual realities (i.e. policies, financial constraints, and biomedical patents) indicated the need to extend principles developed for treatment access and treatment literacy, to support *prevention literacy* and *prevention access* as an integral part of the global response to HIV.

## Introduction

Midway through the fourth decade of the HIV epidemic, there is room for cautious optimism. On World AIDS Day, 1 December 2015, UNAIDS triumphantly announced that 15.8 million people are already accessing life-saving HIV treatment, that new HIV infections have been reduced by 35% since 2000, and that AIDS-related deaths have been reduced by 42% since peaking in 2004 [1]. The World AIDS Day report assured us that we are on the fast track to end AIDS by 2030 as part of the sustainable development goals – though it also cautioned that doing so will require investment, commitment, and innovation [1]. Even with favourable aggregate trends, evidence regarding new infections is mixed when comparing across regions and among diverse populations. In 2014, for example, over 2 million people were newly infected with HIV, 1.2 million people died from AIDS-related causes, and more than 21.1 million people living with HIV still do not have access to life-saving treatment [2].

Before we will be fully justified in claiming that the battle against HIV is soon to be won, we must reckon with the significant challenges that still exist. From the latter part of the third decade to the present, two trends have unfolded that are especially important for thinking about the challenges faced today. First, after years of limited progress in developing new approaches to HIV prevention, we have seen a rapid expansion of the range of available HIV prevention methods, including a new wave of “biomedical” prevention approaches. This, in turn, has led to calls for what has been described as “combination prevention”: the argument that, to be optimally effective, a combination of behavioural, biomedical, and structural/environmental approaches is needed in geographic settings and populations at greatest risk of acquiring and transmitting HIV [3,4]. It has also produced a new emphasis on the increasingly used metaphor, “HIV prevention toolkit” [5]. Yet even with the recognition of a wider range of available possible tools that might be employed for effective HIV prevention, little has been done to articulate pedagogical approaches through which knowledge about these existing tools might best be transmitted – let alone how decisions might be made about which tools to use.

Second, beginning in 2008, we have seen rapidly expanding budgets for the response to HIV begin first to slow, then to plateau, and now, it increasingly appears, to decline in many key sites [3,4]. A combination of donor withdrawal and funding shortfalls for key global health initiatives, such as the Global Fund and PEPFAR, has begun to take place, initiating a new phase in the global response that some worry may soon be understood as “scale-down” in contrast to an earlier period of HIV scale-up [6,7]. On the ground of the global response to HIV, this translates to local-level HIV programmes adjusting to funding cuts by reducing support for prevention interventions based in social and behavioural approaches and instead pinning their hopes on “test and treat” and “treatment as prevention” (or TasP) initiatives. The integration of biomedical prevention and treatment is strategic precisely because treatment is the one programmatic area where budgets cannot be cut without confronting the unacceptable ethical implications of rolling back treatment access [8,9]. Combined with the lack of political support for HIV prevention, a growing treatment gap undermines realistic hope for the end of AIDS [10].

Nonetheless, we find ourselves at a unique moment when we have the widest available range of multimodal techniques to prevent HIV infection. Rather than taking full advantage of these options and scaling-up HIV prevention globally, we appear to be witnessing an unprecedented retreat from commitment to HIV prevention. What we do not know, and what is needed to meaningfully bridge proven biological efficacy to “real-world” effectiveness, is the mechanism through which community education and local ownership can be successfully promoted to make a sustainable impact [11,12].

Thus, to improve implementation and utilization of existing HIV prevention strategies, this article provides necessary contextual and historical reflection to bridge the gap from clinical efficacy to community effectiveness (i.e. knowledge and usage). With this aim in mind, we conducted a critical literature review to (1) articulate a conceptual framework for prevention literacy based on the historical emergence of treatment literacy and the importance of popular pedagogy, and (2) explain the utility of a prevention literacy framework for addressing contextual challenges to realizing effective combination prevention approaches.

## Methods

Seeking to define treatment literacy and health literacy and assess the current need for literacy related to HIV prevention, between January 2015 and October 2015, we conducted a critical global literature review. Given our research question, we were guided by directed content analysis methodological approach, which provided a flexible, non-systematic method for analysing text data through a set of analytic techniques including textual searches, historical reflection, and contextual analyses [13]. Using permutations of the following search terms: “treatment literacy,” “health literacy,” and “prevention literacy,” our review started by searching in electronic databases including MEDLINE, PsycINFO, PubMed, and Google Scholar. As a key component of our methodology, our iterative process of analyses included searches of titles and/or abstracts. Reference lists of retrieved articles were reviewed for additional articles. However, given the dearth of material in peer-reviewed literature about treatment literacy and prevention literacy, the majority of our search was conducted via grey literature, including bilateral and multilateral organizations reports, policy briefs, and targeted website review.

To interpret meaning from the content of text data and to understand the depth and scope of the literature as related to the evolving HIV and AIDS epidemic, our analysis was informed by conceptualizations on distinct social, historical, and behavioural grouping in the three decades of the global response to HIV [14]. Analytic codes were defined prior to search (e.g. prevention literacy, treatment literacy, and health literacy) and during data analysis (e.g. research definition and biomedical paradigm). Literature was primarily reviewed and extracted independently by three trained reviewers. Over a six-month period, routine meetings (approximately twice a month) were conducted with study teams to conduct ongoing content analyses. Differences were resolved through consensus and through discussion with a senior study team member when necessary.

## Results

This review highlighted the range of meanings of “literacy” among key public health agencies (e.g. CDC, NIH, WHO, and UNESCO) and underlined the absence of the conceptual notion of prevention literacy from the peer-reviewed and grey literature (see Table 1). However, these results underscored that the framework for prevention literacy reflects (1) the historical emergence of “treatment literacy” and (2) the importance of popular pedagogy in the global South. Elaboration of these two main themes emergent from our review is presented below and present an interweaving of textual searches, historical reflection, and contextual analyses.

**Table 1:** Definitions of “literacy” within the health arena

*#*	*Illustrative quote*
Treatment literacy	
1	This concept [treatment literacy] denotes not only the capacity of infected persons to use ARVs effectively but also to “interpret information about HIV/AIDS prevention, testing and care” and “prevent HIV/AIDS-related stigma and discrimination” [15].
2	The treatment literacy model, developed by the Treatment Action Campaign (TAC) in South Africa, can be useful for activist groups in working with health professionals… TAC used a “right to health” approach to HIV through a combination of protest, popular mobilisation, and legal action [16].
3	They [TAC] emphasised building capacity among affected people, prioritising those who were disadvantaged, and through the cornerstone concept of treatment literacy. HIV education materials and methods were developed, including on the benefits and side-effects of treatment, embedded in the science of medicine, the political context, human rights, equity of access to health care, and the duties of governments [16].
4	From the outset it [TAC] sought to build a capacity to pursue human rights entitlements directly among the poor and to catalyse a political movement for health. Part of the rationale for this was a distrust of the professional “AIDS and human rights movement,” which often seems part of the global industry spawned by the epidemic, articulate but ineffective [17].
5	Treatment literacy is not taught in a neutral or bio-medical fashion. Information about the science of medicine and health is linked to political science, human rights, equality, and the positive duties on the state [17].
6	Treatment literacy is the base for both self-help and social mobilization. Armed with proper knowledge about HIV, poor people can become their own advocates, personally and socially empowered [17].
7	UNESCO/WHO have defined treatment education as “… forming the bridge between the provision of treatment and the preparation and involvement of people and communities in comprehensive responses to HIV and AIDS” [18].
8	The aim of treatment literacy… was to provide knowledge and skills to understand and manage (to the best of their capacities) their disease, treatment and broader health issues, and to equip them with tools to take some responsibility for their own health [18].
9	Treatment education targeted to individuals and communities encompasses a wide range of ART-related issues, including how the medication should be taken, the importance of adhering to prescribed medication regimens, treatment side effects and how to manage them, interpreting CD4 counts, and how to access local ART services. Treatment education aims to empower persons with HIV to navigate the health system, learn their serostatus, access care, effectively manage ART, and practice HIV transmission-related protective behaviours. In the community, treatment education raises awareness about HIV and AIDS and the effectiveness of ART; encourages people to know their HIV status; and provides information on the availability of VCT and ART, eligibility criteria for accessing ART, and the management of ART. Treatment education also mobilises action to combat AIDS related stigma and discrimination, which act as barriers to accessing HIV counselling and testing – a key entry point for care and treatment, and as barriers to HIV prevention programmes [19].
10	Treatment Preparedness is the term that refers to a person's readiness to begin antiretroviral treatment. It includes “treatment literacy” or having the appropriate knowledge about HIV and the medicines used to treat it, as well as “empowerment” or the meaningful involvement of PLWHA in decisions regarding their care, including the distribution of resources [20].
Health literacy	
11	Health literacy is the degree to which individuals have the capacity to obtain, process, and understand basic health information and services needed to make appropriate health decisions [21].
12	Health literacy has been defined as the cognitive and social skills which determine the motivation and ability of individuals to gain access to, understand and use information in ways which promote and maintain good health. Health Literacy means more than being able to read pamphlets and successfully make appointments. By improving people's access to health information and their capacity to use it effectively, health literacy is critical to empowerment [22].
13	Health Literacy … addresses the environmental, political and social factors that determine health. Health education, in this more comprehensive understanding, aims to influence not only individual lifestyle decisions, but also raises awareness of the determinants of health, and encourages individual and collective actions which may lead to a modification of these determinants. Health education is achieved therefore, through … interaction, participation and critical analysis [22].
14	Health literacy affects people's ability to: Navigate the healthcare system, including filling out complex forms and locating providers and services; share personal information, such as health history, with providers; engage in self-care and chronic-disease management; and understand mathematical concepts such as probability and risk [23].
15	Health literacy incorporates a range of abilities: reading, comprehending, and analyzing information; decoding instructions, symbols, charts, and diagrams; weighing risks and benefits; and, ultimately, making decisions and taking action [24].
Prevention literacy	
16	… In addition to increasing access to treatment, research towards developing a prevention vaccine must be pursued. Innovative models of care were needed to increase retention and improve HIV prevention literacy [25].
17	The Science and Treatment College (STC) seeks to increase the HIV/AIDS science, treatment and prevention literacy of Black Americans in an effort to improve their ability to protect themselves from infection, equip those who are already infected from infecting others, and enhance the community's position as a whole to impact perceptions of and policy regarding HIV/AIDS and health in Black communities [26].
18	Taking structural and environmental challenges into account, some scholars have called for community engagement through “prevention literacy” programs, to address these structural and environmental conditions and to advance the acceptance of and adherence to new modalities of biomedical prevention. Prevention literacy aspires to generate critical knowledge and also ownership of prevention modalities [27].
19	These studies suggest that targeted HIV-prevention interventions can effect improvement for this vulnerable population when programs remain sensitive to gender and cultural differences and expectations, and address the social and economic inequalities that make women vulnerable. Solving these problems on a larger economic scale will require institutional participation and political support for women's equity, HIV prevention literacy, and a broader HIV-prevention agenda [28].
20	We urge international institutions, national governments, and community activists to work together to build demand for HIV prevention: Develop a broad HIV-prevention movement, grounded in the strengthening of natural constituencies for HIV prevention in the communities of those who are most vulnerable and affected; support HIV-prevention literacy at all levels, linked to the successful scaling up of treatment literacy; identify and promote bold advocates and public models for changing harmful social, behavioural, and legal norms and practices; [and] create an active coalition between the movement for HIV prevention and the movement of people living with HIV/AIDS, and link this coalition with other motors of social change, including treatment activists, entrepreneurs, rights activists, and women's and youth activists [11].
21	Treatment and prevention literacy is a community-based activity that helps people learn factual evidence-based information in a non-threatening manner, thus addressing stigma and discrimination, as well as myths about these issues in the community. It is important to engage the community to dispel myths and support changes in how cultures approach prevention so that your health centre can provide effective services [29].

### Historical emergence of treatment literacy: collective agency as central

Our review highlighted “treatment literacy” as a concept and platform for action emerging from treatment activism as a transnational movement. Given the imbalance between need and access, countries in the global South took centre stage in resistance movements to advocate for treatment access. Activists from groups such as the Treatment Action Campaign (TAC) in South Africa advocated for the provision of antiretroviral (ARV) medications for those who needed them, even in low-income societies [16,17,30]. Scholarship underscores that treatment literacy was developed primarily by grassroots community networks in the global South, linking TAC in solidarity with other Southern organizations such as the Lawyer's Collective (India), Global Network of PWA (Thailand), Brazilian Interdisciplinary AIDS Association (ABIA) (Brazil), and international organizations and agencies such as Oxfam and Médecins Sans Frontières (MSF) [31]. The influence of activism in framing treatment literacy highlighted the prominence of treatment access movements on a global scale, which prompted a remarkable surge of international funding that largely characterized the response to HIV between 1999 and 2008 (see Quotes 2 and 6) [5,32].

With the increased provision for antiretroviral therapies (ART) came the increased need for greater understanding and empowered decision-making regarding treatment [33]. While the language of treatment literacy was adopted by the World Health Organization (WHO) and other international health development agencies, the full rights-based implications championed by activist groups were never fully realized by most of these intergovernmental agencies, and a deeper understanding of the true meaning of treatment literacy on the part of the global AIDS policy establishment remains an unfinished project (see Quotes 7, 9, and 10) [17,19,34,35].

Definitions of treatment literacy range across actors, including bilateral and multilateral organizations and scholars (see Quotes 6, 7, 9, and 10). Importantly, textual analysis comparing initial conceptualizations of “treatment literacy” with the later iteration of “health literacy” underscores the loss of collective agency that initially grew out of historically specific struggles for access to HIV treatment (see Quotes 11–15). Activist conceptions of treatment literacy extended far beyond the narrower concept of health literacy and emphasize the political dimension of struggling for access as part of an engaged process of building understanding about a complex and changing range of treatment options. In contrast, the concept of “health literacy” omits the historical grounding and obscures the clear political associations that can be found in relation to the literature that is specifically focused on HIV (see Quote 11). For example, the literature on health literacy focuses primarily on the readability of health information for patients who have limited reading comprehension and/or language barriers when it comes to communicating with healthcare providers (see Quotes 11, 14, and 15) [36].

### Returning to popular pedagogy to co-create prevention literacy

While this critical review identifies the historical context of treatment literacy and more recent call for prevention literacy, these are not mutually exclusive concepts. In contrast to the prevalence of “treatment literacy” and “health literacy,” our results highlight a dearth of material defining and making a case for the relevance of prevention literacy. Though a range of websites used the term “prevention literacy” (see Quotes 16 and 17), unlike “treatment literacy” and “health literacy,” a case has not been made for the importance nor relevance of this concept in improving our response to the HIV epidemic. HIV prevention literacy, as we seek to define it here, parallels the initial intention of treatment literacy, with its emphasis not only on understanding relatively complex technical information about a growing range of prevention methods but also on the question of access as a fundamental human right.

Returning to our findings on “treatment literacy,” critical pedagogy – often described as popular education [37] – emerged as a key driver to the prominence and impact of “treatment literacy” in demanding global equity in HIV treatment access (see Quotes 2, 6, 7, and 9). Importantly, the reviewed literature clearly shows that the process of mobilizing as a community also transforms the social environment of prevention approaches. A critical consciousness approach emerged as contrary to “banking education”-based models that became pervasive in many HIV prevention programmes and health literacy strategies as they later came to be developed by official governmental AIDS programmes and public health “experts” (see Quotes 6 and 10). Furthermore, the banking education model strips students of their agency, as well as their own expertise, and reinforces power asymmetry, establishing a relationship of “oppression” [38]. As an alternative, critical consciousness raising is described as a process in which the student is encouraged to understand and engage dynamically with the instructor to co-create, reinterpret, and apply knowledge [39]. Critical consciousness seeks to draw on the capacities of communities to create the collective agency that is needed to demand the positive change of social constructs that create barriers to social development, health, and well-being. Textual searches and historical reflection highlighted how responses to HIV in community-based settings developed by challenging “scientific expertise,” precisely because of the ways that stigma and discrimination have often been produced and reproduced in scientific discourse and experienced by those living with or at risk of HIV (see Quotes 4 and 19).

Harnessing this historical lesson and the current lack of consensus and use of prevention literacy, today there is a need to develop this concept as not simply the processing of information, but an interactive process of “consciousness raising” and empowerment allowing people to learn, build on existing knowledge, and put their knowledge into action. Success of efforts guided by “treatment literacy,” since the emergence of HIV treatment, has been framed in terms of equity of access, community-based ownership, and multilevel support structures to enable usage and sustainability (see Quote 3 and 4). As such, it is crucial to build and utilize a prevention literacy framework for consciousness raising and engagement, as described by Freire and similar educators. Interventions of the physical environment have focused on creating spaces for discussion and debate – and thus engaging *community-based expertise* – as well as providing the social support needed to enable people to make the choices that are best for themselves [40]. Prevention literacy should entail the ability to process and assess health information to make decisions based on what might be best for each person and builds on popular education approaches to promote activism needed to negotiate and demand the right to these options, and to discuss these decisions with partners and peers [41].

As highlighted by our review, in the policy environment (see Quotes 5, 13, 17, and 18), key sources of community mobilization and engagement posit that health social movements, such as the international women's health movement and the environmental health movement, frame access to prevention and health literacy as political issues that require broader transformations. The grey literature more directly recognizes the fundamental insight that putting knowledge into action may often require advocacy and activism for structural and environmental changes that will be necessary to remove barriers to action (see Quotes 2, 6, 9, 17, and 22). In the case of HIV prevention, activism has historically been crucial to advocating that all people who need it must have equal access to available prevention options as a fundamental part of the human right to health (see Quote 21). As such, we found that by incorporating the historical lessons of the evolution and usage of “literacy” in HIV treatment and prevention efforts, prevention literacy offers the potential framework needed to address physical, economic, social, and policy environments that contextualize HIV vulnerability and challenge the uptake of biomedical prevention methods.

## Discussion

**Figure 1:**
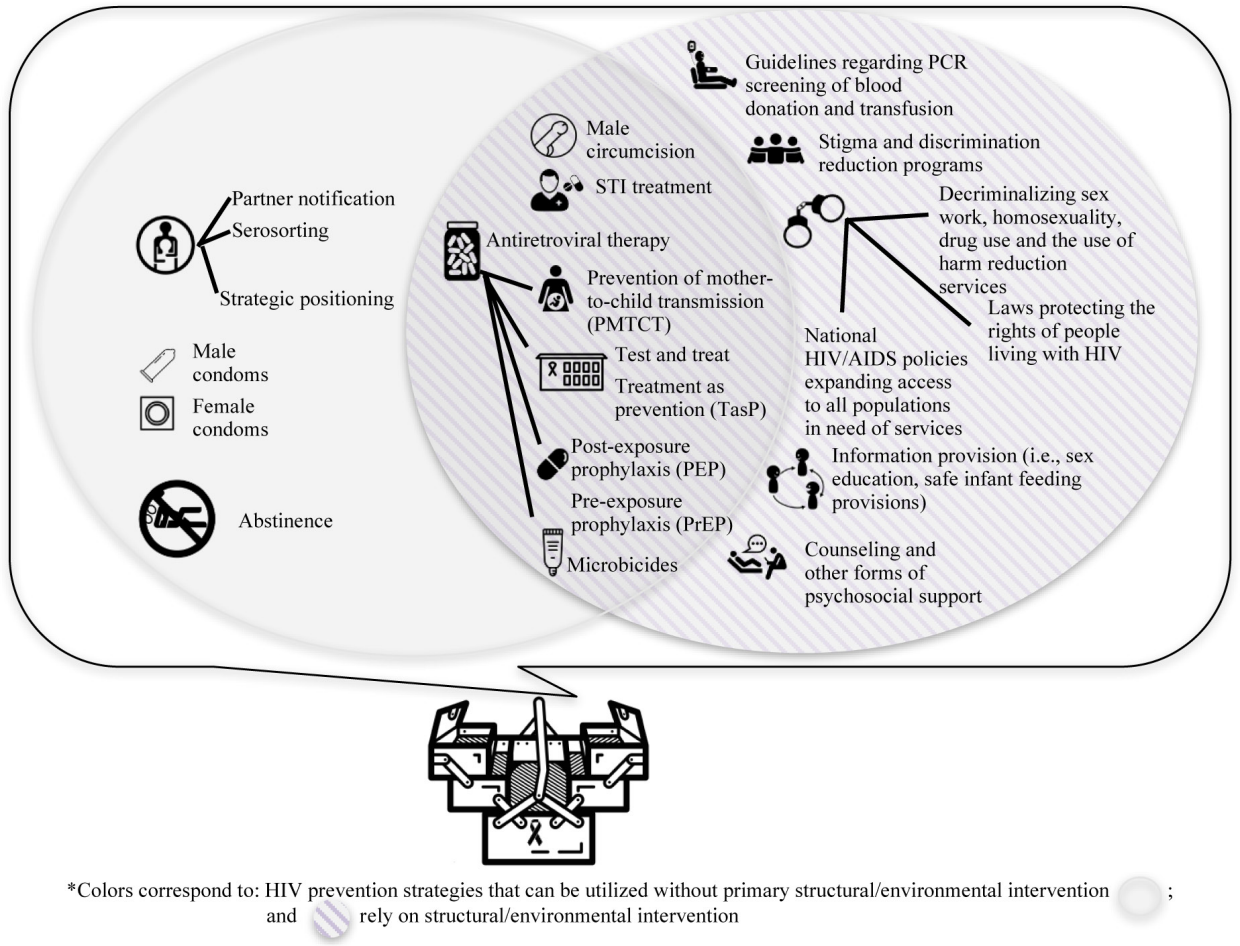
Visualization of existing prevention strategies in the HIV toolkit.

In conceptually mapping the origins of treatment literacy and the relative under-theorization of prevention literacy, our results underscored the importance of the introduction of ART as a major turning point in the global HIV and AIDS epidemic, initially through treatment and, today, in both treatment and prevention efforts. In particular, the past decade has seen a rapid expansion of the uses of ART for HIV prevention, including, preventing mother-to-child transmission [42,43], post-exposure prophylaxis [44], treatment-as-prevention for people living with HIV [45], pre-exposure prophylaxis [46], and topical microbicides [47]. Though the majority of prevention strategies within the biomedical domain are largely indebted to ARTs, strategies such as male and female condoms and voluntary medical male circumcision have also been presented as evidence-based prevention strategies. Importantly, these methods are only part of a larger grouping of HIV prevention approaches including not only biomedical but also behavioural, structural, and environmental approaches. Yet, part of the challenge in today's changing and evolving landscape of prevention strategies is that the dominant biomedical framework (i.e. evidence based) can, at times, obscure the local utility and successful implementation of social and political response efforts (see Figure 1 for a comprehensive visualization of existing HIV prevention strategies).

### Understanding and utilizing the “HIV prevention toolkit”

Understanding the available prevention options is only one piece of the HIV prevention puzzle – assessing their potential impact and the populations they will be relevant for is another. Recognizing HIV as a biological and social disease, evaluation of prevention efforts outside of trial settings has highlighted that, to be effective, biomedical prevention must be coupled with continual behavioural modification and structural support systems [48,49]. Furthermore, existing HIV prevention strategies have a maximum effect when used in combination with each other and adapted to the needs and contexts of specific populations [3,50,51]. This emphasis on combination prevention has further yielded the metaphor of a HIV prevention toolkit promoted by international agenda-setting organizations, such as UNAIDS [6,7].

This metaphor is potentially very useful, as it evokes the image of a process whereby multiple prevention tools are grouped as a joint toolkit from which a single or a combination of tools can be selected for effective HIV prevention. Yet, the notion of a prevention toolkit, at least as it has been conceptualized by agencies such as UNAIDS, has unfortunately been developed and implemented entirely from a top-down or banking education point of view (e.g. the perspective of programme managers, funders, and researchers, primarily from the global North). Reflecting on the historical lessons of “treatment literacy,” counter to the current understanding and utilization of “the prevention toolkit,” the perspective of the users of prevention methods needs to be central.

### Towards a conceptual and pedagogical framework for prevention literacy

These results suggest that to promote effectiveness and sustainability, prevention literacy needs to be grounded in the advancement of human rights and is imperative in the context of HIV prevention because socially marginalized groups often also lack access to preventative medical care. Given the parallels between HIV prevention today and treatment access struggles roughly 15 years ago, historical antecedents may provide valuable lessons. Resource-poor settings seldom prioritize prevention and preventive medicine, and in many places where these services are available, social and cultural barriers (e.g. institutional stigma and discrimination) render them virtually inaccessible. In the absence of a “trustworthy” and supportive healthcare network to acknowledge and address these diverse needs, prevention literacy serves as a viable conduit for marginalized communities to make appropriate choices regarding their prevention options. Access to prevention methods, their understanding, and the empowered choice to use them are universal human rights – essential in order to guarantee the right to health [52]. Similar to the way treatment access and literacy are described, we argue that access to adequate information about the full range of available options and choices in relation to prevention must be every bit as much a human right as access to treatment is now considered [41].

While adopting some components of community-based initiatives, combination prevention and the prevention toolkit, as conceptualized by international agencies, place public health expertise above the knowledge of affected communities. By telling users of HIV prevention what they should be doing, existing prevention pedagogy exemplifies Freire's theory of banking education: seeking to fill up deficit bank accounts with what is deemed by the educator to be “correct” information, rather than collectively constructing knowledge in order to meet the needs of those who will use it. It thus repeats precisely the same kind of errors that have been undermining HIV prevention for decades, ever since we public health experts took over control of prevention methods from affected communities [53].

In part, this drive for a simplified understanding of the range of prevention modalities comes from the increasing dominance of biomedicine, which places an emphasis on efficacy versus effectiveness in HIV prevention strategies [12,54]. It is a product of the tension that exists between *evidence-based* HIV prevention strategies and *real-world* strategies. Whereas evidence-based strategies, heavily emphasized by major AIDS organizations, are determined within the controlled settings of randomized trials, real-world strategies are altered by a myriad of factors that can never be adequately accounted for in the conditions of a controlled trial, such as resource constraints, knowledge, acceptability, and usage. The positioning of new prevention technologies (i.e. the HIV prevention toolkit) as perceived by programme managers fails to account for the social, cultural, and political processes, which to date have been the greatest driver of the HIV epidemic. The use of prevention literacy as a guiding framework can help translate between efficacy and effectiveness to ensure the success of HIV prevention approaches by positioning the expertise of people and communities above biomedical expertise (i.e. reconceiving the HIV prevention toolkit from the point of view of the people who use the tools in their own practice).

Community engagement and advocacy based on prevention literacy also have the potential to buffer against unintended consequences of large scale-up implementation of novel biomedical strategies [11]. For example, literature points to drug misuse related to the potential recreational use of HIV ARV medication within a cocktail of drugs (i.e. *whoonga*) [55] and emergence of social and ethical problems related to the emphasis on male circumcision in the context of a non-circumcising society (e.g. threats to masculinity and virility) [56]. The utility of a prevention literacy framework, raising awareness to methods that do not require partner negotiation, is even more urgent in the context of women [57,58] or young people [59,60] who may not be able to demand or negotiate condom use due to unequal power relations with sexual partners. Many more examples globally point to the urgent need to pair novel prevention technologies with more effective pedagogical approaches to fully utilize and implement available HIV prevention strategies [52,61–63].

Furthermore, the real-world effectiveness of the HIV prevention toolkit hinges on the ability to expand access to prevention methods among the most vulnerable communities and on a global scale. Our review revealed the key finding that treatment literacy, as well as calls for developing prevention literacy, has sprouted from the global South as critical to realizing the promise of biomedical innovation in resource-poor settings [41]. In sub-Saharan Africa, the controversies regarding medication misuse and concealment by participants in Project VOICE (standing for Vaginal and Oral Interventions to Control the Epidemic) offered important global lessons to biomedical researchers regarding assumptions made about significant differences in subjectivity and agency due to nationality and gender [64]. To dispel medical mistrust, a prevention literacy approach promises to generate community ownership of these prevention methods and to create the awareness of historical tensions and experiences of discrimination necessary to mitigate these social and cultural barriers. In resource-scarce settings, with limited health system capacity and diminishing funding for HIV prevention efforts, a prevention literacy framework may generate the level of community ownership necessary to mobilize advocacy networks, target policies to improve access to the prevention toolkit, and generate local empowerment strategies [11,65].

## Conclusions

This critical review highlights that reflecting on historical antecedents may provide valuable lessons given the parallels between HIV prevention today and treatment access struggles roughly 15 years ago; a range of new prevention options have become available, most of which appear to be optimally effective when used in combination. The struggle for “treatment access” reached its zenith in the early-2000s, when the notion of “treatment literacy” emerged from civil society in the global South to empower people living with HIV to demand expanded access to treatment and to be able to use treatments effectively. Our review underscores the loss of collective agency when these concepts became only partially incorporated into the discourse of international agenda-setting institutions.

From these historical antecedents, we argue that given the continued imbalance between need and access, today we also need to both revive and move beyond treatment access and treatment literacy and build *prevention access* and *prevention literacy* as an integral effort to improve the global response to HIV in the 21st century. Existing public health efforts often point to community-based research methods as a way of partnering with “populations,” but this is not enough; recognizing and affirming community-based expertise is crucial to inform and shape the pedagogical approaches and subsequent directives of medical doctors and public health professionals in developing and implementing HIV prevention strategies (which have all too often been ignored or failed to recognize the role of communities in constructing safer practices) [53,66]. Echoing treatment literacy, a core tenet of prevention literacy, needs to be a recognition of existing expertise and a shifting of power to people and communities to make decisions regarding the HIV prevention options that best fit their lived realities.
